# The Magical Horizontal Force Muscle? A Preliminary Study Examining the “Force-Vector” Theory

**DOI:** 10.3390/sports7020030

**Published:** 2019-01-22

**Authors:** David A. Fitzpatrick, Giuseppe Cimadoro, Daniel J. Cleather

**Affiliations:** Research conducted at School of Sport, Health and Applied Science, St. Mary’s University, Twickenham TW1 4SX, UK; davidfitz22@hotmail.com (D.A.F.); giuseppe.cimadoro@stmarys.ac.uk (G.C.)

**Keywords:** hip thrust, vertical jumping, jump training, specificity, dynamic correspondence

## Abstract

The force-vector theory contends that horizontal exercises are more specific to horizontal sports skills. In this context, the focus is on horizontal force production relative to the global coordinate frame. However, according to the principle of dynamic correspondence, the direction of force relative to the athlete is more important, and thus the basis for the force-vector theory is flawed. The purpose of this study was therefore to test the force-vector theory. According to the force-vector theory, hip thrust is a horizontally loaded exercise, and so hip thrust training would be expected to create greater improvements in horizontal jump performance than vertical jump performance. Eleven collegiate female athletes aged 18–24 years completed a 14-week hip thrust training programme. Pre and post testing was used to measure the following: vertical squat jump, vertical countermovement jump, horizontal squat jump, horizontal countermovement jump and hip thrust 3 repetition maximum (3RM). Subjects improved their 3 repetition maximum hip thrust performance by 33.0% (*d* = 1.399, *p* < 0.001, *η*^2^ = 0.784) and their vertical and horizontal jump performance (improvements ranged from 5.4–7.7%; *d* = 0.371–0.477, *p* = 0.004, *η*^2^ = 0.585). However, there were no differences in the magnitude of the improvement between horizontal and vertical jumping (*p* = 0.561, *η*^2^ = 0.035). The results of this study are contrary to the predictions of the force-vector theory. Furthermore, this paper concludes with an analysis of the force-vector theory, presenting the mechanical inconsistencies in the theory. Coaches should use the well established principle of dynamic correspondence in order to assess the mechanical similarity of exercises to sports skills.

## 1. Introduction

Recent years have seen an increase in the popularity of the hip thrust exercise within strength and conditioning practice. The hip thrust is a loaded resistance exercise which utilises the posterior chain hip extensor muscles, such as the glutes and the hamstrings, in order to execute the movement. There are a variety of ways in which the hip thrust can be effectively loaded [1]. The effects of the hip thrust can range from an increase in gluteus maximus size (hypertrophy) to an increase in strength and power [2,3]. There is emerging research that suggests the hip thrust results in training effects that are different to other more traditional approaches, and thus that supports its inclusion in a training programme. For instance, in contrast to the back squat the hip thrust requires a hip extension moment to be sustained even near full hip extension [4] and similarly results in a greater activation of glutes and hamstrings [5].

The increasing prevalence of the hip thrust has in turn led to a number of different research studies that have sought to explore the usefulness of the hip thrust as an exercise to improve sports performance. In particular, there has been interest in evaluating if there are particular physical skills for which the hip thrust is an especially effective training tool [6,7,8]. This can be characterised as an evaluation of the specificity of the hip thrust to various skills. Similarly, the specificity of the hip thrust has been compared to other more traditional resistance training exercises. These recent studies have taken various approaches. For instance, Loturco et al. [8] evaluated the correlation between hip thrust performance and various athletic skills, such as loaded and unloaded vertical jumps, and sprints of varying distances, whereas Contreras et al. [7] considered the effect of a training intervention consisting of hip thrusts on various performance measures, such as vertical jump height, horizontal jump distance, 10 and 20 m sprint times and isometric mid-thigh pull peak force (it should be noted that in both studies the hip thrust was compared to other exercises as well).

In an attempt to explain some of the differences that have been found in studies like those of Loturco et al. [8] and Contreras et al. [7], it has been common to refer to the so-called “force-vector” theory [9,10]. According to this theory, sports skills can be classified on the basis of the direction of force expression relative to the global (world fixed) coordinate frame. Thus sprint acceleration would be considered a horizontal activity, whereas maximum speed running is a vertical activity. Similarly, resistance training exercises would also be classified as horizontal or vertical on the same basis. The force-vector theory then suggests that horizontal exercises are more specific to horizontal skill performance, and vertical exercises more specific to vertical skill performance.

**Figure 1:**
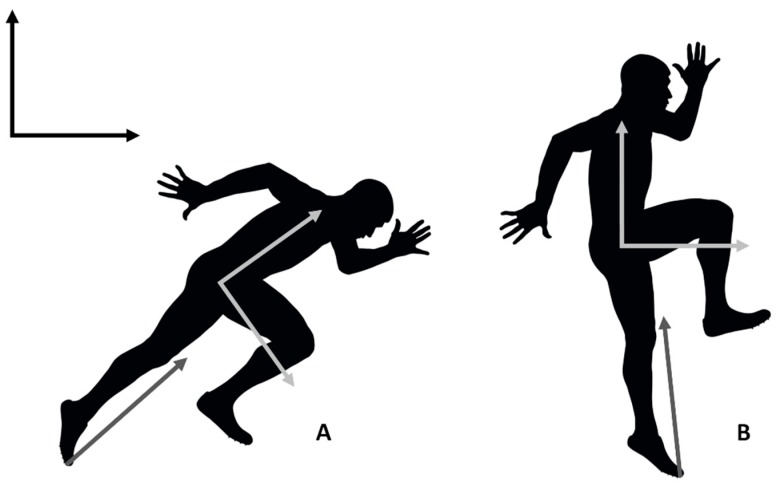
Relationship between global (world fixed—black axes) and local (athlete fixed—light grey axes) coordinate frames. (**A**) An athlete accelerating experiences a ground reaction force (dark grey arrow) which has substantial horizontal and vertical components relative to the global frame. (**B**) If the athlete is rotated such that the local and global frames are aligned, it is apparent that the direction of the ground reaction force relative to the athlete is largely vertical.

In Loturco et al.’s paper [8] they claim that “the force-vector theory is an emergent methodological approach, based on a solid and well established mechanical foundation”. The truth of this statement is hard to evaluate as they do not indicate where this foundation can be found. In fact, we would argue that this theory is actually in direct opposition to the most commonly accepted criteria of mechanical specificity that are used in strength and conditioning; that is, the principle of dynamic correspondence (DC) [11,12]. A fundamental assumption that is made in employing the criteria of DC is that the forces acting on or expressed by the athlete should be considered relative to the local (athlete-fixed) coordinate system of the athlete, not the global frame. For instance, during acceleration and high speed running, the ground reaction forces (GRF) are expressed in a relatively similar direction relative to the athlete. During high speed running, the GRF is predominantly vertical [13,14] (Figure 1B), whereas in acceleration, there is a greater horizontal force relative to the global frame [15] (Figure 1A). However, the reason for this is because the athlete leans forwards in order to project more force horizontally (Figure 1A). The direction of force relative to the athlete is largely the same; for instance, Kugler and Janshen [15] found that there was a strong correlation (*r* = 0.93) between the orientations of the GRF and the body at toe-off. The same is true in horizontal and vertical jumping: the direction of the GRF is similarly relative to the athlete, but in horizontal jumping the athlete leans forwards, meaning that the GRF relative to the global frame is projected more horizontally.

Typically, the criteria of DC are used to support the contention that an activity like back squatting is mechanically similar to an activity like acceleration or horizontal jumping because the direction of the GRF relative to the athlete is similar, despite the fact that the direction of the GRF relative to the global frame might be very different. This is because the direction of force relative to the athlete is what is important in determining their ability to express force. The idea that squatting is less mechanically similar due to the difference in the direction of the GRF relative to the global frame as suggested by the force-vector theory thus directly contradicts the principle of DC.

The purpose of this study was thus to test the force-vector theory by evaluating the effect of a hip thrust training programme on vertical and horizontal jumping performance. Using the terms of the force-vector theory, the hip thrust is a “horizontal” exercise, and thus should produce a greater improvement in horizontal jumping performance than vertical jumping performance. The aim of this study was thus to investigate whether or not there are differences in improvements between horizontal and vertical jumping after a 14-week intervention programme with 11 collegiate female athletes that centred around the hip thrust exercise. It was hypothesised that the horizontal jumps tested would show a more significant improvement than the vertical jumps after the 14-week intervention (in accordance with the force-vector theory). The null hypothesis was that there would be no difference between the improvements in horizontal and vertical jumping performance (as would be suggested by the principle of DC).

## 2. Methods

### 2.1. Experimental Approach to the Problem

A repeated measures within subject design, with no control group, was used for this training study. Each participant carried out a pre-determined hip thrust training programme, performing two sessions per week for 14 weeks. The following variables were measured pre and post training: vertical and horizontal jumps (VJ and HJ), both with and without a countermovement, in addition to 3 repetition maximum (RM) hip thrust strength.

### 2.2. Athletes

In order to be eligible for this study, all subjects were required to be female collegiate athletes who were at least 18 years of age. They also had to have at least one year of experience in a sport that involved both horizontal and vertical jumping, such as soccer, basketball and rugby. Athletes also needed to be completely injury free. Fifteen athletes volunteered to take part in the study; however, two withdrew based on time restraints, one had a scheduling conflict and one left due to an illness (unrelated to the study). Therefore, 11 athletes successfully completed the study (Table 1). The subjects were all recreational athletes competing at the college level. The athletes had 5–12 years experience competing in their sport and 1–2 years of strength and conditioning exposure. The study was approved by the Ethics Sub-Committee of St. Mary’s University, Twickenham and athletes provided written informed consent prior to taking part.

### 2.3. Procedures

#### Pre-Testing Session

The pre-testing session began with each athlete filling out the necessary paperwork (Par-Q, Consent form). Following this, athletes listened to a brief presentation outlining the details of what the training session would involve, as well as an overview of the 14-week programme. This was then followed by a 10 min dynamic warm up, the jump tests and finally finished with the 3RM hip thrust strength test. Details for each of these tests are provided below.

### 2.4. Dynamic Warm Up

The 10 min dynamic warm-up targeted the lower body. It consisted of a range of mobility drills with particular focus on the hips and lower back. This was followed by glute activation work involving mini bands, in exercises such as clam shells and crab walks. This warm up process was used before the pre and post testing sessions, and before each of the 28 sessions throughout the training study.

### 2.5. Jump Tests

Two different jump tasks (VJ and HJ) were performed with (CMJ) and without (SJ) a countermovement (arm swing was permitted). The order of the jump tasks was randomised first, and then the order of condition (SJ or CMJ) was randomised for the task. The vertical jumps were measured using a timing mat (Probotics Vertical Jump Mat, Huntsville, AL, USA) and the horizontal jumps were measured using a standing long jump mat (Atreq standing long jump mat 3.5 m, Dewsdbury, UK). The testing involved 12 jumps in total–6 for each task broken into 3 SJ and 3 CMJ. With jumps taking less than 5 s to complete, a 30 s rest period was given between each jump, giving a work to rest ratio of at least 1:6. Technical execution of the jumps was visually inspected by the lead researcher, and if an incorrect technique was perceived (e.g., use of countermovement during the SJ condition) then the jump was repeated. The athlete’s best jump from each format was recorded for the study.

### 2.6. Hip Thrust 3RM

Hip thrust performance was evaluated by testing 3RM, as this measure was used in a previous study that evaluated the effect of a hip thrust training programme, thus facilitating a more direct comparison [7]. The authors of the previous study [7] cited safety concerns as their reason for preferring 3RM over 1RM testing. The athletes used the following hip thrust technique throughout testing as well as during the 14-week programme. They began by sitting on the floor beside a padded bench or box. The bench was aligned with the mid region of the shoulder blades and the barbell was set up with large plates, technique or bumper plates, to lift the bar off the ground. Then the athletes straightened the legs out and rolled the bar in over the crease of their hips, bending the legs until a 90 degree angle relative to the knee and the ground was achieved with a vertical tibia. The athlete was in a seated position with the knees bent and the feet placed shoulder width apart. In order to provide a comfortable lifting experience, a barbell pad or cushioning of some sort, such as a towel or a mat, was placed between the barbell and the hip bone.

From this position, the athletes were instructed to take a deep breath, brace the core and lift through the hip extensor muscles. The athletes were advised to keep the spine in a neutral position and not hyperextend during the uplift, while simultaneously keeping the knees over the toes, not allowing them to cave in. From here, the athletes pushed down into the ground through their heels, driving the hips up. The back then hinged up onto the bench with the hips rising until the hips and torso were parallel with the ground while the feet were kept flat. Meanwhile, the lead researcher observed the exercise closely and advised the athlete to not go into hyperextension at this position of the exercise. The top level was held for one second, then the hips were slowly lowered back down to the floor, in a controlled and safe manner. The neck and head were kept in alignment with the spine in order to ensure maximum safety, enabling the exercise to be executed in a controlled manner throughout the movement. The athletes were instructed to lower until approximately 90° of hip flexion and compliance with this instruction was monitored by the lead researcher.

The athletes followed the process below in order to ascertain their 3RM.

Warm-Up Set One: 10 repetitions @ 30 kg
Warm-Up Set 2: 5 repetitions @ 10–20% increase of previous weight used
Trial Set One: 3 repetitions @ 20–30% increase of previous weight used
Trial Set Two (and beyond if necessary): 3 repetitions @ 10–20% increase of previous weight used

A successful trial was achieved when the athlete’s hips reached parallel to the floor. For each successful trial, 10–20% of the previous weight was added until the athlete performed an unsuccessful attempt. After an unsuccessful effort, one more trial was attempted with a 5–10% reduction from the previous weight. The percentage of weight added was determined by the difficulty or ease at which the participant carried out the previous set.

### 2.7. 14-Week Resistance Training Programme

Each session was primarily based around 6 hip thrust sets (Table 2). In addition to these sessions, some upper body and core exercises were included. Details of the training sessions are shown in Table 3. The hip thrust programme consisted of two 7-week cycles, during which intensity was gradually incremented concurrent with a decrease in volume (a traditional linear periodization). The lead researcher found this approach to programming the hip thrust to be effective in his previous practice.

### 2.8. Post-Testing

Following the completion of the 14-week training programme, post study testing was carried out by the study leader. This session was identical in format to the pre-testing session, apart from the completion of the pre-testing paperwork.

#### Statistical Analysis

All data was stored and analysed using IBM SPSS (Statistical Package for Social Studies, Version 24, International Business Machines Corp., New Orchard Road, Armonk, NY, USA). Intra-class correlation coefficients (ICCs) and coefficients of variations were calculated. The reliability indicated by the ICCs was interpreted as follows: <0.50 = poor, 0.50–0.75 = moderate, 0.75–0.90 = good, >0.90 = excellent [16]. Data was considered to have low variance if the coefficient of variance was less than 1. A factorial repeated measures analysis of variance (time × jump task × countermovement) was used to determine if there was any significant difference between the pre and post measures of the variables tested. Partial eta squared values (*η*^2^) were calculated as a measure of effect size. In addition, the standardized difference between pre- and post-test scores was calculated using Cohen’s *d*, and the size of the effect was interpreted as follows: 0.2 = small, 0.5 = medium, 0.8 = large [17]. A significance level for all analyses was set at *p* < 0.05 a priori. All vertical jump performances were scaled by dividing each performance by the mean performance of the group for the pre-test vertical countermovement jump. Similarly, all horizontal jump performances were scaled by dividing each performance by the mean performance of the group for the pre-test horizontal countermovement jump. This then allowed the vertical and horizontal jumps to be compared on a like-to-like basis.

## 3. Results

All athletes attended each of the required sessions, giving a 100% attendance rate. Athletes had an average weekly volume of 3324 ± 196 kg in the hip thrust during the course of the training programme.

The coefficients of variation for the jump trials ranged between 0.119 and 0.177 (Table 4). The ICCs comparing the with and without countermovement conditions were between 0.899 and 0.951. The ICCs for the pre- to post-test comparison were between 0.832 and 0.895.

The athletes’ improvement in hip thrust performance was statistically significant (33.0%, *p* < 0.001, *η*^2^ = 0.784; Table 5). Similarly, there was a statistically significant improvement in all measures of jump performance (time effect—*p* = 0.004, *η*^2^ = 0.585), but there was no statistically significant difference in the improvement in vertical or horizontal jump performance (time × jump task effect—*p* = 0.561, *η*^2^ = 0.035).

## 4. Discussion

The results presented above illustrate that the training was effective and that the athletes experienced a substantial increase in their hip thrust and jumping performance. However, the study did not support the hypothesis that horizontal jumping performance would improve to a greater extent than that of vertical jumping as would be suggested by the force-vector theory. Instead, there were no differences in the changes in horizontal and vertical jumping performances. This instead provides support for the importance of considering the direction of force relative to the athlete, not the global frame, as is suggested by the principle of dynamic correspondence.

In a recent paper, Vigotsky and colleagues [18] asked whether we “have lost the ‘mechanics’ in ‘biomechanics’?”. We would argue that the force-vector theory is a very clear example of such a phenomenon, as it is based upon several “mechanical misconceptions”. The most significant problem with the force-vector theory is that it gives primacy to the direction of force relative to the global frame, whereas what is most important is the direction relative to the athlete [11]. Of course, if an athlete wants to move horizontally, relative to the global frame, they need to direct force horizontally. However, it is important to understand that an athlete’s primary strategy for doing this is to change their own orientation (which involves complex coordination strategies). Consider Figure 1: the direction of force relative to the athlete is approximately vertical; however, by leaning forward, they direct a proportion of this force horizontally. For instance, Kugler and Janshen [15] have shown that the direction of the GRF is highly correlated (*r* = 0.93) to the angle of body lean. This is not to say that an athlete can’t steer the direction of the force that they are expressing somewhat. For instance, Hof [19] has suggested that one function of the biarticular muscles is to facilitate the steering of the force on the pedal during cycling. However, the effect of this is small relative to the primary strategy.

The classification of exercises by proponents of the force-vector theory is also inconsistent. For instance, the hip thrust is classified as a horizontal exercise on the basis that the GRF is directed horizontally, relative to the athlete (note that this is often described as acting antero-posteriorly; that is, using language that describes directions relative to the athlete). However, in classifying an activity like acceleration or horizontal jumping, supporters of the force-vector theory consider the direction of the GRF relative to the global frame. Again, this shows a fundamental misunderstanding of the mechanics: one can’t argue that the direction of two vectors is similar if the directions are evaluated in two different coordinate frames that are rotated relative to one another.

A final problem with the force-vector theory is that the mechanism by which a given exercise is more specific is unclear. Even if we accept the contention that training with “horizontal” exercises improves the athlete’s ability to direct force horizontally relative to the global frame, how is this achieved if it is not simply by changing the orientation of the athlete relative to the global frame? In the case of the hip thrust, how does performing an exercise with the knee flexed at 90° improve the ability of the athlete to steer the GRF during closed kinetic chain leg extension? That is, in an entirely different posture to the one that is used in the sport skill.

It is not our intent to argue that there are not differences in the training effects of hip thrusts and more traditional exercises, or that the hip thrust is not more specific to certain activities. Rather, there are other perfectly viable explanations for the results of Contreras et al. [7] that are in accord with the principle of DC and do not require the invocation of the force-vector theory. For instance, Bezodis and colleagues [4] have shown that in contrast to the back squat, the hip extensor moment required to complete a hip thrust is still relatively high as the hip approaches full extension. The hip thrust thus requires force to be expressed in a different range of motion to the back squat. According to the second criterion of DC, the region of accentuated force production, an exercise is more mechanically specific if force is produced in a similar range to the sport skill. The evidence of Bezodis and colleagues would therefore suggest that on the second criterion of DC, the hip thrust may be more specific to any skill which requires substantial force expression when the hip is near full extension. Contreras and colleagues [20] have also argued that different hip extension exercises activate the hip musculature in different ranges of motion. This provides a rationale for using a range of different exercises. Finally, Contreras et al. [5] have shown that glute and hamstring activation is higher in the hip thrust than the back squat. Again, this provides a potential explanation as to why the hip thrust might be more specific to skills that are relatively more hip dominant.

There are a number of limitations to this work. In particular, a timing mat was used for the assessment of vertical jump height, due to the availability of this equipment. Previous work has shown that timing mats may produce greater jump heights than other methods [21]. However, because the same equipment was used for pre- and post-tests, the effect of this was minimized. Similarly, this study had no control group which reduces our ability to draw conclusions about the absolute efficacy of the training approach employed here.

## 5. Practical Applications

The purpose of this article is not to suggest that the hip thrust is not a performance enhancing exercise. Instead, the results of this article suggest that the hip thrust was an effective tool for enhancing jump performance in a population of female collegiate athletes, and thus this study does provide some support for the use of the hip thrust to enhance performance. It should be noted however, that this study had no control or comparison group, and so our ability to give recommendations as to the efficacy of specific exercises is limited. What this article does demonstrate though is that the well established principle of DC should be the coach’s go-to when evaluating the specificity of exercises. Similarity, it is important to analyse the direction of force relative to the athlete, not the global frame, when considering specificity. Coaches should recognise that the force-vector theory is a result of confused mechanical reasoning and is not a principle that should guide their practice.

## Figures and Tables

**Table 1:** Athlete Characteristics.

N	Age (years)	Height (cm)	Weight (kg)
11	22 ± 2	165.4 ± 4.5	69.6 ± 12.2

**Table 2:** Hip Thrust Programme Layout.

Week	Sets	Reps	Load
1	6	12	40% of 3RM
2	6	12	45% of 3RM
3	6	12	50% of 3RM
4	6	10	55% of 3RM
5	6	10	60% of 3RM
6	6	8	65% of 3RM
7	6	8	70% of 3RM
8	6	12	45% of 3RM
9	6	12	50% of 3RM
10	6	10	55% of 3RM
11	6	10	60% of 3RM
12	6	8	65% of 3RM
13	6	8	70% of 3RM
14	6	8	75% of 3RM

**Table 3:** Layout of each workout session during programme.

Process	Day One	Day Two
1. Warm-up	10 min warm up	10 min warm up
2. Hip thrust	6 sets of hip thrusts	6 sets of hip thrusts
3. Upper body—One exercise from the group	Incline Press or Military Press or Bench Press 4 sets × 8 reps each	Incline Press or Military Press or Bench Press 4 sets × 45 s
4. Upper body—One exercise from the group	Bent over row or bench pull or seated row 4 sets × 8 reps each	Bent over row or bench pull or seated row 4 sets × 45 s
5. Core work	Abdominal and/or lower back work 4 sets × 45 s	Abdominal and/or lower back work 4 sets × 45 s

**Table 4:** Intra-class correlation coefficients (ICCs) and coefficients of variation (CV). ICCs are presented with 95% confidence intervals given in parentheses. The first two columns represent the comparison of the with and without countermovement conditions. The third column is the comparison from pre- to post-test.

Jump Type	ICC			CV	
	Pre	Post	Pre–Post	Pre	Post
Vertical jump (m)					
With countermovement	0.913 (0.710–0.976)	0.934 (0.775–0.982)	0.878 (0.611–0.966)	0.177	0.137
Without countermovement			0.895 (0.659–0.971)	0.170	0.138
Horizontal jump (m)					
With countermovement	0.899 (0.670–0.972)	0.951 (0.830–0.987)	0.857 (0.555–0.959)	0.119	0.129
Without countermovement			0.832 (0.492–0.952)	0.140	0.134

**Table 5:** Pre- and post-test performance measures. All measures showed a statistically significant (*p* < 0.05) improvement from pre to post test.

Measure	Pre	Post	Change (%)	Cohen’s *d*
Vertical jump (m)				
With countermovement	0.39 ± 0.07	0.42 ± 0.06	+5.95	0.371
Without countermovement	0.39 ± 0.07	0.42 ± 0.06	+7.67	0.477
Horizontal jump (m)				
With countermovement	1.47 ± 0.18	1.55 ± 0.20	+5.95	0.462
Without countermovement	1.49 ± 0.21	1.57 ± 0.21	+5.42	0.388
Hip thrust 3RM (kg)	98.0 ± 10.8	130.2 ± 20.7	+32.95	1.399
